# *Borrelia burgdorferi* Infection Is Worth Screening to Investigate Sensorineural Hearing Loss Etiology: A Systematic Review

**DOI:** 10.1002/hsr2.70974

**Published:** 2025-06-30

**Authors:** Abhinav Bhattarai, Sangam Shah, Madhur Bhattarai, Garima Dhakal, Sunraj Tharu, Mandira Khadka, Prakash Sharma, Arun Kharel, Basanta Sharma Paudel, Prativa Subedi, Shyam Kumar Mishra

**Affiliations:** ^1^ Institute of Medicine Tribhuvan University Kathmandu Nepal; ^2^ Medical Database Irvine California USA; ^3^ Maharajgunj Nursing Campus Tribhuvan University Kathmandu Nepal; ^4^ Kist Medical College Lalitpur Nepal; ^5^ Department of Microbiology, Institute of Medicine Tribhuvan University Kathmandu Nepal; ^6^ School of Optometry and Vision Science University of New South Wales Sydney Australia

**Keywords:** *B. burgdorferi*, sensorineural hearing loss, SNHL

## Abstract

**Background and Aim:**

Sensorineural hearing loss (SNHL) is a common hearing disorder prevalent. *Borrelia burgdorferi* (*B. burgdorferi*) is a spirochete whose infection has been shown to result in SNHL. This systematic review aims to investigate the prevalence and association of *B. burgdorferi* infection in SNHL.

**Methods:**

A systematic literature search on the databases Medline, Google Scholar, and UpToDate was performed. The study selection process was done in accordance with the PRISMA guideline. In brief, studies were selected first by title and abstract screening followed by a full‐text inspection. The study was included if the study reported the incidence of *B. burgdorferi* infection in patients with SNHL. The quality assessment of the included studies was performed using the Joanna Briggs Institute Critical Appraisal tool. Data on study characteristics, patient demographics, audiological, microbiological, symptomatological, and therapeutical findings were extracted.

**Results:**

The study search retrieved a total of 8772 studies and 9 of them met out eligibility requirement. There were altogether 964 SNHL patients. In total, 71 (7.3%) were tested positive for *B. burgdorferi* infection. The commonest symptoms in infected SNHL patients were tinnitus (53%) and vertigo (47%). Patients treated with steroids along with ceftriaxone showed a higher hearing recovery rate.

**Conclusion:**

*B. burgdorferi* infection is prevalent among patients with SNHL and should be investigated whenever no other reasons of hearing loss are established.

**Abbreviations**
ELISA: enzyme‐linked immunosorbent assay
OspC: outer surface protein
PCR: polymerase chain reaction
PRISMA: Preferred Reporting Items for Systematic Reviews and Meta‐analysis
SNHL: sensorineural hearing loss

## 1 Introduction

Sensorineural hearing loss (SNHL) refers to the loss of auditory perception due to any pathology in the cochlea, or auditory nerves. SNHL is the most common hearing loss prevalent with 5–27/100,000 individuals being affected every year [1]. SNHL is diagnosed when the individual has a hearing disability of 30 dB for at least three successive frequencies [2]. SNHL can affect either one or both ears. One of the challenges for otologists in dealing with SNHL is establishing the etiology. Frequent causes of SNHL include upper respiratory tract infections, noise‐induced, head trauma, Meniere's disease, drug‐induced ototoxicity, gene mutations, and microcirculatory disturbances [3]. Lyme disease is one of the risk factors to develop SNHL [4]. However, in around 10%–15% of SNHL cases, the etiology remains unknown. Such cases are dealt with as idiopathic SNHL. Recently, numerous studies have been done and several pieces of evidence have been generated on the microbial origin of SNHL [5].

*Borrelia burgdorferi* (*B. burgdorferi*), first identified in the early 1980s, is a spirochete bacteria known to cause Lyme disease in humans. It is a tick‐borne disease that spreads via a tick, *Ixodes scapularis*, also called the black‐legged tick [6]. *B. burgdorferi* is the commonest causative organism of tick‐borne disease in North America, with an incidence of 25,000–30,000/year. The highest incidence has been reported in children between 5 and 9 years old and adults between 45 and 59 years old. Males are shown to be affected more frequently as compared to females [7]. Infection is acquired by the tick's attachment to human skin for at least 24 h. *B. burgdorferi* spreads on the skin and then to the viscera by hematogenous and lymphatic routes. Infection can persist from several weeks to months until the immune system takes over the pathogen [8].

Untreated *B. burgdorferi* infection is shown to result in neurologic sequelae [9]. Numerous studies have discussed the association of *B. burgdorferi* with SNHL. Studies have reported a substantial proportion of SNHL patients being infected with *B. burgdorferi* [10, 11, 12, 13, 14, 15, 16, 17, 18]. However, in routine practice, *B. burgdorferi* infection screening is not generally performed while diagnosing and treating SNHL patients. It is indeed concerning that screening of this particular pathogen is often neglected by otologists and the cases are reported as idiopathic. Importantly, studies have emphasized the necessity of antimicrobial therapy in SNHL patients with *B*. *burgdorferi* infection, which can affect patient prognosis and recovery [10, 13]. Currently, the question is whether *B. burgdorferi* infection should be taken into account while finding the cause of SNHL or not. This is the first systematic review that aims to answer this question through a thorough investigation of studies previously performed in this context and disseminate the integrative findings that will aid otologists having to deal with SNHL in clinical practice.

## 2 Methods

### 2.1 Search Strategy and Study Selection

We performed the systematic review based on the Preferred Reporting Items for Systematic Reviews and Meta‐analysis (PRISMA) guideline [19]. Two authors G. D. and S. T. conducted a systematic literature search on the databases Medline, Google Scholar, and UpToDate for contextual studies published until October 10, 2022. A search string was created using search terms “*Borrelia burgdorferi*,” “spirochetes,” “spirochetes,” “Lyme disease,” “Hearing,” “Auditory,” “Otologic*,” “Deaf*,” “Hearing impairment,” “SSNHL,” “SNHL,” and “sensorineural hearing loss” connected by “AND” and “OR” Boolean operators.

The retrieved results were exported into a CSV file and duplicates were screened and removed both automatically and manually. The primary screening of studies was done by inspection of titles and abstracts. Studies with relevant information were sorted for full‐text inspection. Full‐text inspection of all shortlisted studies was carried out by author A. B. based on the eligibility criteria. The references of those studies selected for review were further checked to obtain more studies relevant to the context. Any discrepancies during the selection process were resolved by discussion with author S.S.

### 2.2 Eligibility Criteria

We included all studies that possessed the following eligibility criteria:

1. Studies that reported the incidence of *B. burgdorferi* infection in patients with SNHL.
2. Studies whose full text was available in English.

We excluded case reports, editorials, abstract presentations, commentaries, and review articles.

### 2.3 Data Extraction

Authors G. D. and S. T. extracted the data from selected studies onto a prespecified Microsoft Excel data extraction sheet. The data extraction sheet was designed to store information on demographic, audiological, microbiological, symptomatological, and therapeutical data. The demographic data extracted were: (1) author, (2) published year, (3) study country, (4) population size, (5) population age distribution, and (6) population gender distribution. Similarly, the audiological information extracted were: (1) number of SNHL patients, (2) type of onset, (3) ear involvement, and (4) diagnosis of SNHL. The following microbiological data were extracted: (1) number of SNHL patients with *B. burgdorferi* infection, (2) diagnostic methodology of infection, and (3) the antigens/antibodies tested. Likewise, the symptoms of infected SNHL patients were also extracted. Finally, the data extracted on therapeutics were: (1) drugs administered for hearing recovery, (2) antibiotics administered, and (4) the number of recovered and not recovered patients.

### 2.4 Quality Assessment of the Included Studies

The risk of bias was assessed using the Joanna Briggs Institute Critical Appraisal tool [20]. It consists of eight domains: (1) inclusion criteria clearly defined, (2) study subjects and setting described, (3) ascertainment of exposure, (4) standard criteria for measurement, (5) confounding factor identification, (6) strategy to deal with confounding factors, (7) outcome measurement, and (8) appropriate statistical analysis. The full text of each included study was critically analyzed and the bias domains were answered either “yes,” “no,” “unclear,” or “not applicable.”

### 2.5 Outcome of Interest

The primary outcome of interest of this systematic review is to investigate the association of *B. burgdorferi* infection in SNHL patients. Secondary outcomes of interest include: assessing the methodology of SNHL and infection diagnosis, symptoms, treatment, and recovery.

### 2.6 Data Synthesis

Discrete data were summarized using percentages or ratios. Mean, standard deviation, median, and interquartile range were used to summarize continuous data.

## 3 Results

### 3.1 Study Search and Study Selection

**Figure 1:**
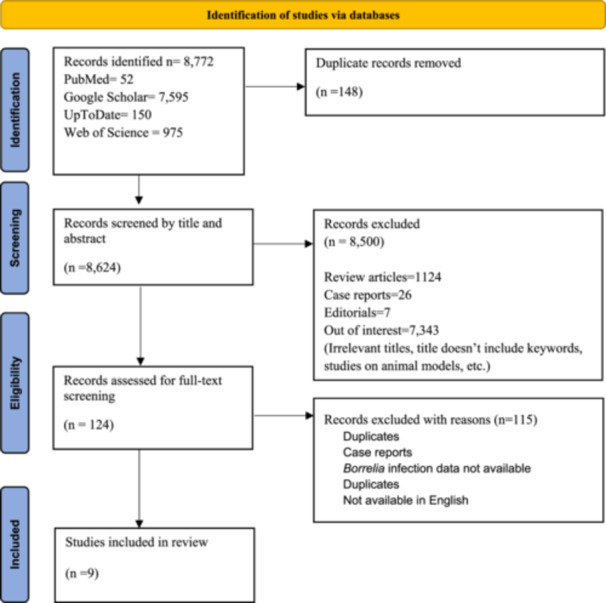
PRISMA flowchart describing the study selection.

The literature search retrieved a total of 8772 studies. After screening by titles and abstracts, 124 studies were subjected to full‐text screening. Finally, nine studies were included after the full‐text screening. The detailed study selection process is displayed in the PRISMA flowchart in Figure 1.

### 3.2 Quality Assessment of the Included Studies

The detailed results of the quality assessment of the included studies are shown in Table 1. No studies were excluded based on the results of the quality assessment.

**Table 1:** Quality assessment results of the included studies.

References	Bias domains							
	Inclusion criteria clearly defined	Study subjects and setting described	Ascertainment of exposure	Standard criteria for measurement	Confounding factor identification	Strategy to deal with confounding factors	Outcome measurement	Appropriate statistical analysis
Sowula et al. [10]	Yes	Yes	No	Yes	No	No	Yes	Yes
Lorenzi et al. [11]	Yes	Yes	Yes	Yes	Unclear	Unclear	No	Yes
Finizia et al. [12]	Yes	Yes	Yes	Yes	No	No	Yes	Unclear
Peltomaa et al. [13]	Yes	Yes	Yes	Yes	Yes	Yes	Yes	Yes
Hanner et al. [14]	Yes	Unclear	Yes	Yes	No	No	Yes	Unclear
Hyden et al. [15]	Yes	Unclear	Yes	Yes	Yes	Yes	Unclear	No
Abuzeid et al. [16]	Yes	Yes	No	Yes	No	No	No	No
Gagnebin et al. [17]	Yes	Yes	No	Yes	No	No	No	No
Richardson et al. [18]	Yes	Unclear	No	Yes	Unclear	Unclear	No	No

### 3.3 Descriptive Characteristics of the Included Studies

A total of nine studies were included in our study. Seven studies were from Europe while one each were from North and South America. All of them were cohort studies. The study population size ranged from 19 to 230. Altogether, there were 964 patients with SNHL. The mean age of the population ranged from 43 to 55 years. The male and female populations were unequally distributed. The descriptive characteristics of the included studies are displayed in detail in Table 2.

**Table 2:** Descriptive characteristics of the included studies.

S. N.	References	Publication year	Study country	Population size	Population age	Sample (hearing + infected) gender
1	Sowula et al. [10]	2021	Poland	86	Male: 47.3 (31–59) Female: 48.3 (29–62)	M:F = 1:2
2	Lorenzi et al. [11]	2009	Brazil	47	42 ± 13	M:F = 2:3
3	Finizia et al. [12]	2009	Sweden	19	50.4	M:F = 13:6
4	Peltomaa et al. [13]	2000	Finland	230	43	M:F = 13:33
5	Hanner et al. [14]	1989	Sweden	98	54.8 (33–80)	M:F = 8:9
6	Hyden et al. [15]	1995	Sweden	37	54.7 (32–77)	NR
7	Abuzeid et al. [16]	2008	USA	181	55.6 (15–88)	M:F = 87:94
8	Gagnebin et al. [17]	2002	Switzerland	182	45 (15–82)	M:F = 99:83
9	Richardson et al. [18]	1994	UK	100	51 (11–92)	M:F = 56:44

### 3.4 Patient Characteristics: Audiology and Microbiology

**Figure 2:**
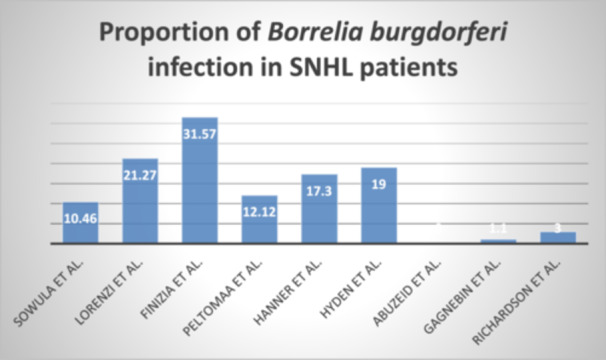
Bar chart showing the proportion of SNHL patients infected with *Borrelia burgdorferi*.

**Figure 3:**
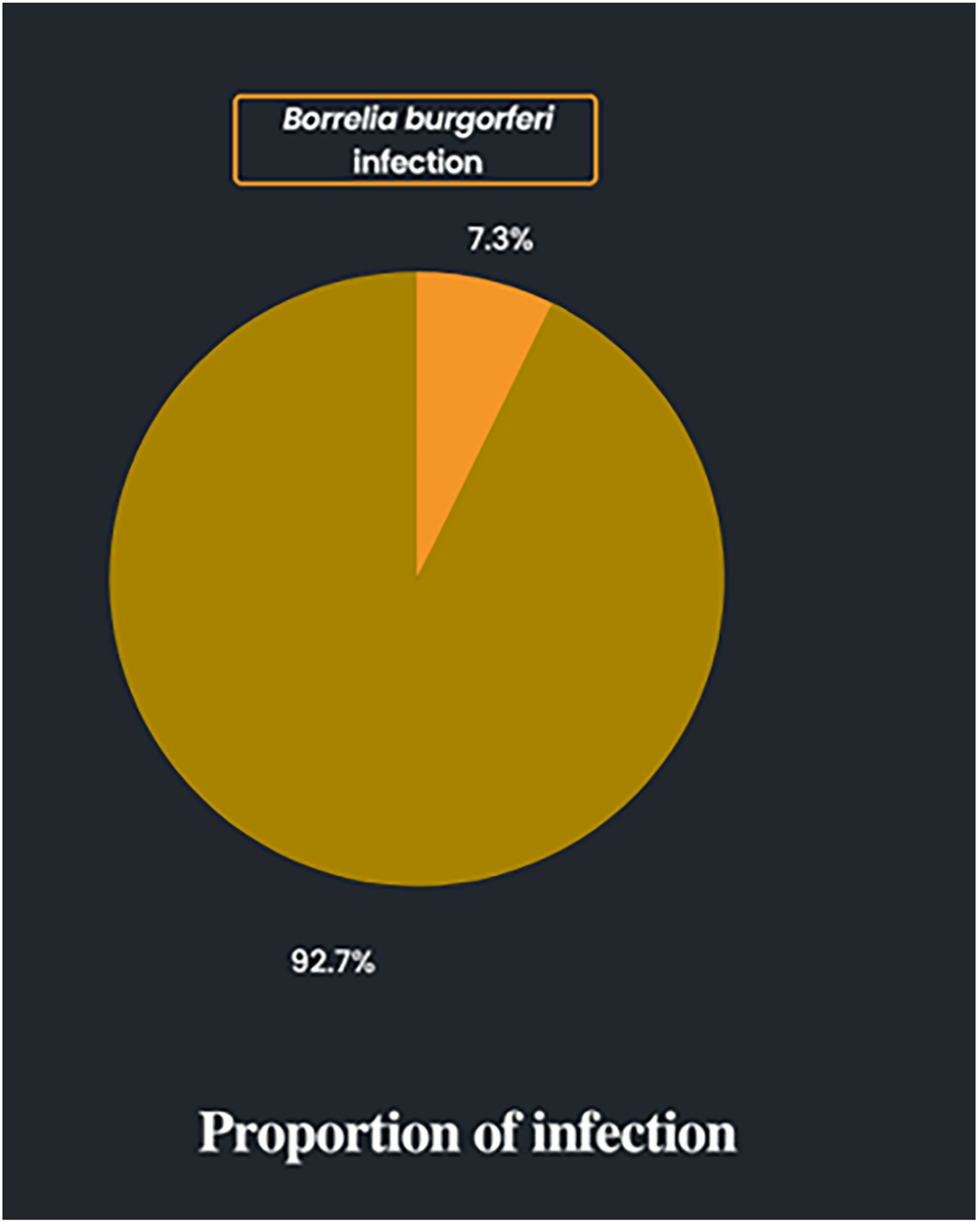
Proportion of *Borrelia burgdorferi* infection in SNHL.

The audiological and microbiological profiles of the study population are displayed in Table 3. The patients were diagnosed with SNHL by pure‐tone audiometry analysis under frequencies ranging from 0.125 to 8 kHz. In total, 71 out of 964 patients with SNHL had active or past infection with *B*. *burgdorferi*. In most studies, infection was diagnosed using enzyme‐linked immunosorbent assay (ELISA). Few studies utilized a more confirmatory test, the western blot, and examined for the presence of *Borrelia* proteins. One study utilized indirect immunofluorescence and another also tested for *B. burgdorferi* antigens. Figure 2 shows the proportion of patients with SNHL that had *B. burgdorferi* infection. A majority of infected patients presented with sudden hearing loss. Three studies reported a progressive hearing loss as well. Both unilateral and bilateral hearing losses were reported. Figure 3 summarizes the proportion of *B. burgdorferi* infection in SNHL.

**Table 3:** Audiological and microbiological profile of the study population.

References	SNHL patients	Onset/ear involvement	Methodology diagnosis of hearing loss	SNHL patients with *Borrelia* infection	Methodology for *Borrelia* detection	Antigens/antibodies tested
Sowula et al. [10]	86	Sudden/unilateral	Pure‐tone audiometry (250, 500, 1000, 2000, 4000, 6000 Hz)	9 (10.46%)	Screening by ELISA, then confirmation by western blot	Antigens: outer surface protein (OspC), p100, VlsE Antibodies: IgG+ = 4 IgM+ 7 Both+ = 2
Lorenzi et al. [11]	47	Sudden/unilateral	Pure‐tone audiometry (250, 500, 1000, 2000, 4000, 6000 Hz)	10 (21.27%)	Screening by ELISA, then confirmation by western blot	Antibodies IgM+ = 3 IgG+ = 7
Finizia et al. [12]	19	Sudden/unilateral	Pure‐tone audiometry (500, 1000, 2000, 4000 Hz)	6 (31.57%)	ELISA	Antibodies IgM+ = 1 IgG+ = 5
Peltomaa et al. [13]	230	Sudden/unilateral, bilateral	Pure‐tone audiometry (125, 250, 500, 1000, 2000, 4000, 8000 Hz)	20 (12.12%)	ELISA and PCR	Antibodies IgM+ = 9 IgG+ = 9 Both+ = 2
Hanner et al. [14]	98	Sudden/unilateral	Pure‐tone audiometry (125, 2000, 4000, 8000 Hz)	17 (17.3%)	Indirect immunofluorescence	Antibodies IgG = 17
Hyden et al. [15]	21	Sudden/unilateral	NA	4 (19%)	NR	Antibodies IgM = 2 IgG = 2
Abuzeid et al. [16]	181	Sudden, progressive/unilateral, bilateral	Pure‐tone audiometry (250, 500, 1000, 2000, 3000, 4000, 6000, 8000 Hz)	0 (0%)	Screening by ELISA, then confirmation by western blot	NA
Gagnebin et al. [17]	182	Sudden, progressive/unilateral, bilateral	NA	2 (1.1%)	Screening by ELISA, then confirmation by western blot	NA
Richardson et al. [18]	100	Sudden, progressive/unilateral, bilateral	NA	3 (3%)	Screening by ELISA, then confirmation by western blot	NA

Abbreviations: ELISA, enzyme‐linked immunosorbent assay; NA, not available; OspC, outer surface protein; PCR, polymerase chain reaction.

### 3.5 Patient Characteristics: Symptomatology and Treatment Outcomes

Tinnitus and vertigo were present in most of the SNHL patients infected with *B. burgdorferi* (53% and 47%, respectively). Figure 3 shows the distribution of symptoms in SNHL patients with infection. These patients were treated for both hearing loss and *B. burgdorferi* infection. The hearing loss in most studies was managed by steroid therapy using prednisone, prednisolone, and dexamethasone. Infection was treated with ceftriaxone, doxycycline, or penicillin. In the study by Sowula et al. [10], people treated with corticosteroid in combination with ceftriaxone rather than doxycycline showed greater recovery. The doses of the antibiotics were 200 mg/day and 2 g/day for doxycycline and ceftriaxone, respectively. Out of four recovered patients, three received ceftriaxone while one received doxycycline. Few studies have been treated using penicillin. Table 4 discusses the symptoms and treatment in SNHL patients infected with *B. burgdorferi*.

**Table 4:** Table showing the symptoms and treatment outcomes in SNHL patients with infection.

References	Symptoms in patients (hearing + infection)	Drug(s) administered for hearing recovery	Antibiotic(s) administered	Recovered	Not recovered
Sowula et al. [10]	Tinnitus (5/9 = 55.55%) Vertigo (3/9 = 33.33%)	Corticosteroids (dosage NR)	Doxycycline (200 mg/day) Ceftriaxone (2 g/day)	Total = 4 Doxycycline = 1 Ceftriaxone = 3	Total = 3 Doxycycline = 3 Two patients lost follow‐up
Lorenzi et al. [11]	Tinnitus (9/10 = 90%) Vertigo (7/10 = 70%)	Dextran, dexamethasone, papaverine, vitamin A (dosage NR)	Ceftriaxone (1 g/day) Amoxicillin (1.5 g/day)	NR	NR
Finizia et al. [12]	NR	Prednisolone (30 mg) or betamethasone (3 g) or dextran (250–500 mL)	Tetracycline hydrochloride (200 mg)	47.2%	NR
Peltomaa et al. [13]	Tinnitus (16/20 = 80%) Vertigo (10/20 = 50%)	Corticotrophin‐releasing hormone, prednisone (1 mg/kg)	Ceftriaxone (100 mg/kg/day)	17	3
Hanner et al. [14]	Vertigo (15/17 = 88.23%) Facial palsy (3/17 = 17.64%)	NR	Benzylpenicillin (30 mg/kg)	5	8
Hyden et al. [15]	NR	NR	Penicillin	16	4 (one lost follow‐up)
Abuzeid et al. [16]	Vertigo (141/181 = 77.9%)	NR	NR	NR	NR
Gagnebin et al. [17]	NR	NR	NR	NR	NR
Richardson et al. [18]	NR	NR	Penicillin 500 mg	0	3

Abbreviation: NR, not reported.

## 4 Discussion

This systematic review aimed to investigate the prevalence of *B. burgdorferi* infection in patients with SNHL and find out the association via a deep literature review. Our study included a total of 964 patients diagnosed with SNHL, and 71 of them tested positive for *B. burgdorferi* infection. Based on the data from nine studies, we found that the pooled prevalence of *B. burgdorferi* infection in SNHL is 7.3%, which is notably not negligible. The prevalence ranged as high as 31.57% in the study of Finizia et al. [12] to as low as 0% in the study of Abuzeid et al. [16]. All in all, rates of *B. burgdorferi* infection varied but did exist in a nonignorable fashion.

The prevalence of infection does differ based on the weather and geography of the study country which affects the concentration of ticks, tick seasonality, and their habitat, and this is the possible reason behind the variation in the prevalence of *B. burgdorferi* infection in the studies that we included. A study found that the life‐cycle completion of the black‐legged tick and the genotypic distribution of *B. burgdorferi* were heterogeneous based on seasonal climate patterns [21]. The study further put forward that the host of *B. burgdorferi* is highly active in July–September and in temperatures above 18°C. This might be the reason behind the difference in the prevalence of infection as seen in our study.

The possible mechanism behind the cause of hearing loss in *B. burgdorferi* infection has been linked to consequent inflammatory, immunologic, neurologic, and microangiopathic events, however, the exact mechanism is unclear [22]. One possible reason might be due to the elevation of fibrinogen during the hematological dissemination of the pathogen due to which the cochlear blood flow is altered [23]. Several studies have shown a direct association of SNHL with cochlear microcirculatory disturbances [24, 25, 26, 27, 28]. Few studies have also proposed in vivo cross‐reaction of *B. burgdorferi* antibodies to neuronal tissues and this might also be the reason behind the compromise of auditory nerve function.

Additionally, while discussing the immunological cross‐reaction, in vitro cross‐reaction in *B. burgdorferi* antibodies has led to doubts about the sensitivity and specificity of immunological detection assays [29]. False IgM positivity and a seronegative window period are considered important pitfalls while diagnosing infection. According to Leeflang et al. [30], the sensitivity of commercial Lyme disease detection kit is ~54% during erythema migrans, 81% during neuroborreliosis, 96% during Lyme arthritis, and 97% in acrodermatitis chronica atrophicans. Moreover, the heterogeneous species of *Borrelia* circulating in Europe has mandated the use of mixtures of antigens in detection kits which is a potential invitation to further cross‐reactions and false seropositivity. Therefore, otologists should not entirely rely on ELISA reports and should consider the clinical as well as the epidemiological profile of SNHL patients, above all, otologists should be acquainted with the possibility of SNHL by *B. burgdorferi* infection, instead of assigning an idiopathic reason.

Our study holds both strengths and limitations. This is the first systematic review performed to assess the prevalence and association of *B. burgdorferi* infection and SNHL. We included only SNHL patients and assessed the methodology of disease diagnosis. We further put down the symptomatology and therapeutics of the included studies. Our study, however, holds some limitations. First, the population size was limited and a majority of them belonged to similar geographical and climatic locations. Due to the loss of follow‐up in the course of the treatment in included studies, we could not integrate the treatment outcomes into our findings. Likewise, statistical testing by meta‐analysis for significant prevalence could not be performed due to heterogeneous populations and potential confounders. Lastly, other sources of information, such as unpublished sources, and gray literature were not searched which might have created a potential omission of relevant studies.

## 5 Conclusion

*B. burgdorferi* infection is prevalent among patients with SNHL and should be investigated whenever no other reasons for hearing loss are established. Infected patients who develop SNHL experience tinnitus as well as vertigo. Administration of ceftriaxone in combination with steroids can aid in a better prognosis and hearing recovery.

## Author Contributions

**Abhinav Bhattarai:** conceptualization, writing – original draft, methodology. **Sangam Shah:** conceptualization, writing – original draft, methodology. **Madhur Bhattarai:** methodology, writing – original draft, data curation. **Garima Dhakal:** methodology, data curation; formal analysis. **Sunraj Tharu:** methodology, data curation, formal analysis. **Mandira Khadka:** data curation, formal analysis, writing – review and editing. **Prakash Sharma:** writing – review and editing. **Arun Kharel:** writing – review and editing. **Basanta Sharma Paudel:** writing – review and editing. **Prativa Subedi:** writing – review and editing. **Shyam Kumar Mishra:** writing – review and editing. All authors have read and approved the final version of the manuscript.

## Ethics Statement

This was a meta‐analysis, so ethical approval was not required for the study.

## Conflicts of Interest

The authors declare no conflicts of interest.

## Transparency Statement

The lead author Abhinav Bhattarai affirms that this manuscript is an honest, accurate, and transparent account of the study being reported; that no important aspects of the study have been omitted; and that any discrepancies from the study as planned (and, if relevant, registered) have been explained.

## Data Availability

Abhinav Bhattarai had full access to all of the data in this study and takes complete responsibility for the integrity of the data and the accuracy of the data analysis. No new data were generated in the study.
